# Triple Cytokine FluoroSpot Analysis of Human Antigen-Specific IFN-γ, IL-17A and IL-22 Responses

**DOI:** 10.3390/cells3041116

**Published:** 2014-11-27

**Authors:** Tomas Dillenbeck, Eva Gelius, Jenny Fohlstedt, Niklas Ahlborg

**Affiliations:** 1 Mabtech AB, Box 1233, SE-131 28 Nacka Strand, Sweden; E-Mails: eva@mabtech.com (E.G.); jenny.fohlstedt@mabtech.com (J.F.); niklas@mabtech.com (N.A.); 2 Department of Immunology, Stockholm University, SE-106 91 Stockholm, Sweden

**Keywords:** FluoroSpot, ELISpot, T-helper cell subsets, IFN-γ, IL-17A, IL-22, Th1, Th17, Th22

## Abstract

The involvement of T-helper (Th)1, Th17 and Th22 cell subsets, in immunity, as well as in pathological inflammatory reactions, makes it important to determine their relative proportion. A triple FluoroSpot detecting the hallmark cytokines of Th1 (IFN-γ), Th17 (IL-17A) and Th22 (IL-22) was developed and evaluated using human peripheral blood mononuclear cells from healthy donors incubated with tetanus toxoid, *Candida albicans* extract, mycobacterial purified protein derivative or medium only. Antigen stimulation yielded mainly cells secreting IFN-γ, IL-17A or IL-22 alone but lower proportions of double-secreting cells were also found; triple-secreting cells were rare. The response to *C. albicans* contrasted in that higher proportions of IL-17A single secreting as well as co-secreting cells, in particular IL-17A/IL-22, were found. The FluoroSpot analysis correlated well with single cytokine ELISpot assays ran in parallel and the methods displayed a comparable sensitivity. The results demonstrate the functionality of the FluoroSpot assay for simultaneous analysis of distinct Th1, Th17, Th22 as well as intermediate cell populations. The method provides a mean for a simple and rapid analysis of the involvement of these cells in immunity and disease.

## 1. Introduction

Antigen activation drives CD4+ T cells into various effector subsets with different or overlapping roles in immune responses and inflammatory processes. The effects displayed by CD4+ T-helper (Th)-cell subsets are to a large extent determined by their cytokine profile. Two T-cell subtypes early defined were Th1 and Th2 cells, with Th1 cells primarily producing IFN-γ and not IL-4 and *vice versa* for Th2 cells [1]. More recently T-cell subsets with other distinct cytokine expression patterns have been defined, including Th17 cells that principally secrete IL-17A [2] and Th22 cells that are defined by the production of IL-22 in the absence of any of the hallmark cytokines of Th1, Th2 and Th17 cells [3].

Th1, Th17 and Th22 cells are all involved in immune responses to pathogens. Th1 cells are vital for the activation of cytotoxic T-cells, regulation of B-cell responses, activation of macrophages and protection against intracellular pathogens in particular [4]. Th17 cells are involved in immunity against extracellular pathogens including *Candida albicans* and *Staphylococcus aureus* [5,6] and Th22 cells, due to their expression of skin-homing receptors, appear to be recruited to the skin for tissue repair and protection against pathogens [7].

Aberrant activation of these T-cell subtypes is associated with increased susceptibility to various pathogens, autoimmunity and inflammatory reactions involved in these diseases. Heightened activity of Th1 cells has been associated with pathogenic inflammatory reactions in for example autoimmune diseases [8]. More recent findings suggest that also Th17 are involved in inflammatory processes in autoimmune diseases and skin disorders [9,10] and several studies have implicated Th22 involvement in dermal inflammation and skin disorders including psoriasis and atopic and allergic dermatitis [11,12,13].

Although Th1, Th17 and Th22 cells have been associated with many diseases, their relative importance for pathogenesis remains to be elucidated. One important aspect is the correlation between the frequency of each T-cell subset and disease activity; hence methods facilitating the enumeration of Th1, Th17, Th22 as well as intermediate T-cell subtypes are important.

One of the most sensitive assays for enumeration of cytokine-secreting T cells is the Enzyme-Linked ImmunoSpot (ELISpot) assay. It is a robust and versatile assay that can be applied to many different analytes, although it is limited in that it is restricted to detection of a single cytokine. Dual color ELISpot utilizing two different enzymes generating substrate products of different colors has been used [14,15], but the results can be ambiguous if one of the precipitating substrate products obscures the other. The FluoroSpot assay overcomes this limitation by utilizing fluorophores for the detection of multiple cytokines [16] and also facilitates analysis of more than two cytokines [17]. By use of selective filters for excitation and emission, fluorescent signals in FluoroSpot can be cleanly separated and individual images of each fluorophore captured, void of interference and bleed-through artifacts. Individual analysis of each analyte is therefore possible, much like a series of separate single color ELISpot assays. Double- and triple-stained spots are then identified based on the spot positions on the different filter images.

The aim of this study was to develop and evaluate a triple cytokine FluoroSpot capable of enumerating IFN-γ-, IL-17A-, and IL-22-secreting cells, as well as potential intermediate populations secreting mixtures of these cytokines. The cells analyzed were human peripheral blood mononuclear cells (PBMC) *ex vivo* stimulated with antigens previously shown to elicit IFN-γ, IL-17A and/or IL-22 secretion, including *C. albicans* extract (CA), tetanus toxoid (TT) and mycobacterial purified protein derivative (PPD).

## 2. Experimental Section

### 2.1. Human PBMC

Buffy coats from anonymous regular blood donors were obtained from the Blood Central at Karolinska University Hospital. PBMC were prepared using Ficoll-paque density centrifugation and were frozen at −80 °C in 20% fetal calf serum (FCS) and 10% dimethyl sulfoxide. The cells were kept frozen in liquid nitrogen until used. PBMC concentration and viability (>85%) were determined using the Guava ViaCount assay (Guava Technologies, Hayward, CA, USA).

### 2.2. Assay Reagents and Stimuli

RPMI 1640, HEPES, penicillin/streptomycin, and low endotoxin FCS were purchased from Invitrogen Life Technologies (Carlsbad, CA, USA). The serum lot was pretested to check for adverse effects. All ELISpot reagents: mAbs 1-D1K, 7-B6-1-biotin, MT12A3, MT7B27-biotin, MT44.6, MT504-biotin, streptavidin-alkaline phosphatase (SA-ALP), and 5-bromo-4-chloro-3-indolyl phosphate/nitro-blue tetrazolium (BCIP/NBT) and FluoroSpot reagents: mAbs 1-D1K, 7-B6-1-FS-FITC, MT12A3, MT7B27-biotin, MT44.6, MT504-BAM, 13A5, 39C3-BAM, IL1β-I, IL1β-II-biotin, anti-FITC-490, anti-BAM-490, SA-550, anti-BAM-640, and fluorescence enhancer were from Mabtech AB, Nacka Strand, Sweden. 96-well MSIP PVDF membrane ELISpot plates (Cat. Num. S5EMO77I10) and low fluorescent 96-well PVDF membrane FluoroSpot plates (Cat. Num. S5EJ104I07) were obtained from Millipore, Bedford, MA, USA. TT was purchased from Statens Serum Institute, Copenhagen, Denmark, PPD from Apoteket, Stockholm, Sweden, CA from Greer, Lenoir, NC, USA, LPS from Sigma-Aldrich, Stockholm, Sweden, and R848 from Enzo Life Sciences, Farmingdale, New York, NY, USA.

### 2.3. IFN-γ/IL-17A/IL-22 FluoroSpot

Low fluorescent 96-well PVDF plates were pre-wetted with 15 µL 35% ethanol for no more than 1 min, immediately followed by washing with sterile water five times (200 µL/well). 100 µL of anti-IFN-γ (1-D1K), anti-IL-17A (MT44.6) and/or anti-IL-22 (MT12A3), all diluted to 15 µg/mL in sterile PBS, were added to each well and incubated overnight at 4 °C. The following day plates were washed five times with sterile PBS (200 µL/well), and blocked with 200 µL/well cell culture medium (RPMI 1640 supplemented with 10% heat-inactivated FCS, 1 mM glutamine, 100 units/mL penicillin, 100 µg/mL streptomycin and 0.5 mM HEPES) for at least 1 h. The blocking medium was removed and fresh medium with or without stimuli (20 µg/mL of CA, 10 Lf/mL of TT or 5 µg/mL of PPD) added to each well. Rested PBMC were added to each well (300,000 cells/well), each sample and condition analyzed in triplicates, and incubated for 44 h at 37 °C and 5% CO_2_. Cells were removed by washing the plates five times with PBS (200 µL/well) in an automated ELISA washer (Bio-Tek Instruments Inc., Winooski, VT, USA), and 100 µL of anti-IFN-γ (7-B6-1-FS-FITC; 1:200), anti-IL-17A (MT504-BAM; 1:200) and/or anti-IL-22 (MT7B27-biotin; 0.5 µg/mL) in PBS with 0.1% bovine serum albumin (PBS/BSA) was added to each well. Plates were incubated for 2 h at RT, followed by washing as described above. Secondary detection reagents (anti-FITC-490, anti-BAM-640, and/or SA-550) were diluted 1:200 in PBS/BSA and 100 µL added to each well for a 1 h incubation at RT. Plates were washed as above and 50 µL fluorescence enhancer added to each well for 15 min. The enhancer was discarded thoroughly, the plate underdrain removed, and the plates left to dry protected from light. Plates were read and analyzed in an ELISpot/FluoroSpot reader system (iSpot Spectrum, AID, Strassberg, Germany) with software version 7.0, build 14790, where fluorescent spots were counted utilizing separate filters for FITC, Cy3, and Cy5. Camera settings (exposure and gain) were adapted for each filter to obtain high quality spot images preventing over- or under-exposure.

### 2.4. Monocyte FluoroSpot (IL-6/IL-1β)

Low fluorescent PVDF plates were pre-wetted and washed as described and coated with 100 µL of 15 µg/mL anti-IL-6 (mAb 13A5) and 15 µg/mL anti-IL-1β (mAb IL1β-I) in sterile PBS. Plates were incubated, washed, and blocked as describe above. Two thousand five hundred PBMC were added to each well, with our without stimuli (20 µg/mL CA extract, 10 µg/mL TT, 5 µg/mL PPD, 1 µg/mL R848, or 50 ng/mL LPS) and incubated overnight at 37 °C and 5% CO_2_. The following day plates were washed and 100 µL PBS/BSA with anti-IL-6 (mAb 39C3-BAM, diluted 1:200) and anti-IL-1β (mAb IL1β-II-biotin, diluted to 1 µg/mL) added to each well. Plates were washed as above, and secondary detection reagents anti-BAM-490 and SA-550 (diluted 1:200 in PBS/BSA) added to all wells. Plates were then washed, treated with fluorescence enhancer, and read as described above.

### 2.5. ELISpot

Sterile 96-well PVDF plates were ethanol treated as above, followed by the addition of 100 µL/well of mAb to IFN-γ (1-D1K), IL-17A (MT44.6) or IL-22 (MT12A3), all diluted to 15 µg/mL in sterile PBS. Plates were incubated at 4 °C overnight, washed the following day, and cells +/− stimuli added as above. After 44 h incubation at 37 °C and 5% CO_2_, cells were washed away with PBS in an automated ELISA washer as above, and mAb to IFN-γ (7-B6-1-biotin; 1 µg/mL), IL-17A (MT504-biotin; 1 µg/mL), or IL-22 (MT7B27-biotin; 0.5 µg/mL) in 100 µL PBS/BSA added to each well. Plates were incubated for 2 h at RT, followed by washing as above. SA-ALP was diluted 1:1000 in PBS/BSA and added to all wells and incubated for 1 h at RT. Plates were again washed, and spots developed with BCIP/NBT substrate for 10 min (stopped by extensive washing in tap water). The plate underdrain was removed and the plates left to dry before reading and analysis in an ELISpot/FluoroSpot reader system (iSpot Spectrum).

### 2.6. Statistics

Statistical analyses were performed using Analyse-it (Analyse-it Software Ltd, Leeds, UK). The correlation between the cytokine responses assessed by different methods was analyzed using Spearman’s rank order correlation coefficient r_s_. The difference in the proportion of dual- and triple-spots induced by different antigens was analyzed by Wilcoxon rank sum test. *p* < 0.05 was considered statistically significant.

## 3. Results and Discussion

### 3.1. Design and Analysis of the FluoroSpot Assay

**Figure 1:**
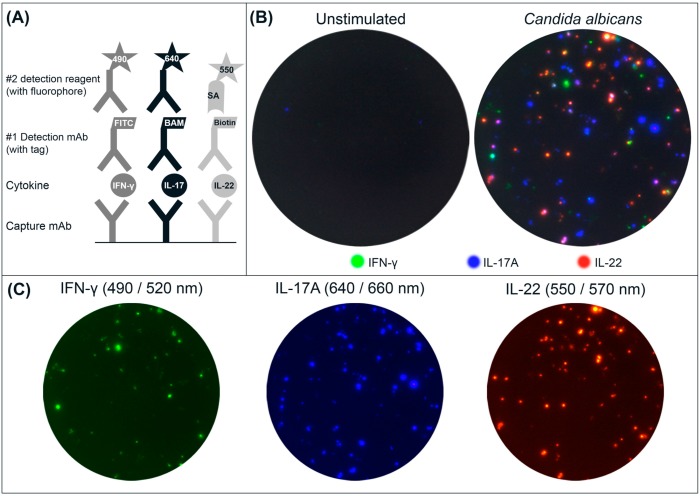
(**A**) Schematic drawing of the triple IFN-γ/IL-17A/IL-22 FluoroSpot assay. Capture mAbs to the cytokines are coated in the same well. After cell incubation, each cytokine is detected by the respective detection mAb labeled with a unique tag enabling the binding of secondary detection reagents with fluorophores. The tag/anti-tag systems used were FITC/anti-FITC-mAb (IFN-γ), peptide BAM/anti-BAM-mAb (IL-17A) and biotin/Streptavidin (IL-22). Cells secreting single cytokines will be detected by one detection system alone, whereas cells secreting multiple cytokines will be detected by two or three detection mAbs and result in double- or triple-stained spots respectively; (**B**) Example of image overlays from wells with unstimulated and CA-stimulated PBMC (300,000 cells/well); (**C**) Individual images were captured for IFN-γ (FITC filter), IL-17A (Cy5 filter), and IL-22 (Cy3 filter) and used to generate the computerized overlay (shown for CA-stimulated cells).

A triple FluoroSpot assay based on mAbs to human IFN-γ, IL-17A and IL-22 was developed. Detection was performed in two steps to amplify the signals. The respective detection mAbs were labeled with unique tags that in turn were bound by secondary anti-tag reagents conjugated with fluorophores absorbing and emitting light at 490/520 nm (IFN-γ), 640/660 nm (IL-17A) and 550/570 nm (IL-22) (Figure 1A).

PBMC were incubated, with or without stimuli, and secreted cytokines were detected. Following that, the plates were analyzed in a FluoroSpot reader equipped with filters for both incoming and outgoing light to avoid bleeding over between the fluorophores. An example of spots obtained with PBMC incubated with CA or with medium only is shown in Figure 1B. Spot colors shown are not true to each fluorophore’s respective emission wavelength, but have been substituted to yield a more intuitive visual representation. The change of colors has no impact on the analysis of spots [18]. Spots representing seven different secretion patterns can be found: three for single-stained spots, three for the combinations of double-stained spots, and one for triple-stained spots. The number of single, double and triple spots is determined by comparing spot locations on each filter image. Spots with matching coordinates in two or three filter images are defined as double and triple spots respectively.

### 3.2. Comparison of FluoroSpot and ELISpot

**Figure 2:**
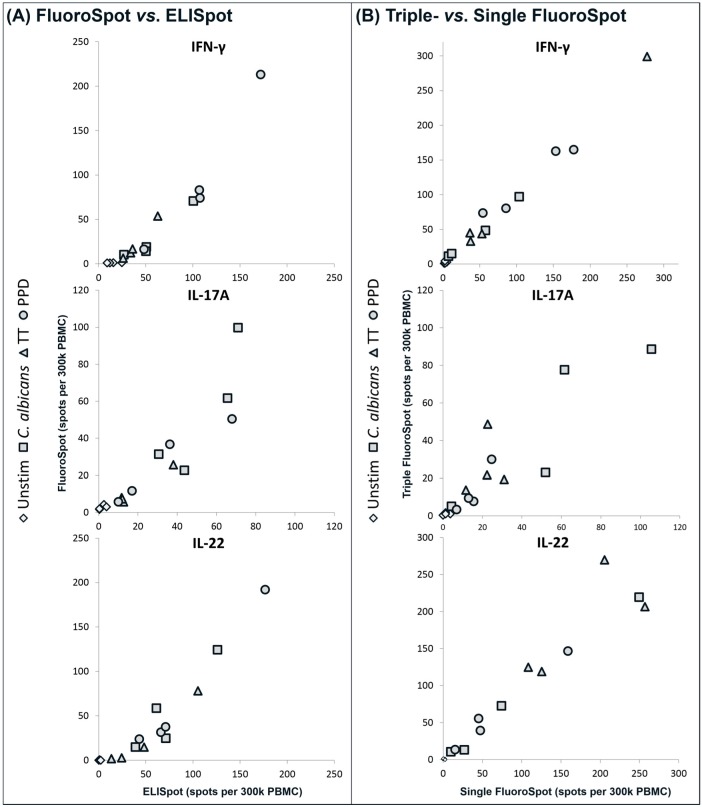
(**A**) Comparison of single cytokine FluoroSpot *versus* ELISpot. PBMC from four donors were stimulated with CA, TT or PPD or incubated with medium only and the number of cells secreting IFN-γ, IL-17A or IL-22 was determined by FluoroSpot and ELISpot; (**B**) Comparison of triple *versus* single cytokine FluoroSpot. PBMC from four other donors than in (A) were cultured as in (A) and analyzed in triple and single FluoroSpot for the number of cells secreting IFN-γ, IL-17A or IL-22. To obtain comparable data, spots in the triple FluoroSpot were counted as single cytokine spots, *i.e.*, double and triple positive spots were counted as positive for each cytokine. The symbols in (A) and (B) represent the mean of triplicates with different symbols corresponding to unstimulated or antigen-stimulated PBMC. The variation within triplicates for the antigen-stimulated wells was on average 25%. All comparisons in (A) and (B) correlated positively (r_s_ = 0.92–0.98 and *p* < 0.0001). The experiments were repeated with PBMC from additional donors with similar results.

The detection sensitivity obtained in the FluoroSpot was compared to ELISpot. Since the ELISpot measures single cytokine-producing cells, the FluoroSpot used was a single cytokine assay (Figure 2A). Spot numbers obtained by the two methods correlated well and the total spot counts for antigen-induced responses were comparable. However, ELISpot yielded somewhat higher spot counts for the unstimulated cells (Figure 2A).

### 3.3. Evaluation of Potential Absorption Effects

In order to assess if the presence of multiple capture mAbs in one well had any impact on the production of the other cytokines, the triple FluoroSpot assay was compared to single FluoroSpot assays for all three cytokines (Figure 2B). A good correlation between the assays was seen and hence no apparent absorption effects caused by the combination of three capture mAbs were observed.

### 3.4. Cytokine Profile of Antigen-Specific Responses

**Figure 3:**
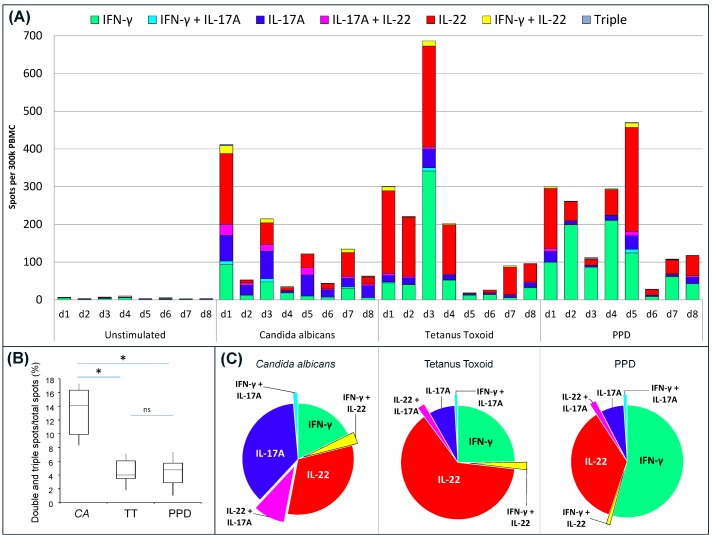
IFN-γ/IL-17A/IL-22 triple FluoroSpot analysis of PBMC responses to CA, TT and PPD. PBMC incubated with stimuli or medium alone were analyzed for spots representing cells secreting single, double and triple combinations of IFN-γ, IL-17A and IL-22. (**A**) The bars represent individual responses (n = 8) to all antigens divided into single, double or triple spots; (**B**) Analysis of the proportion of double- and triple-stained spots induced by the different antigens shown for all eight donors. The box plot show median (line in box), min and max (vertical lines), and 25 and 75 percentiles (box) for CA, TT, and PPD stimulation (n = 8). Statistical significance is indicated as * (*p* < 0.01) or ns (not significant); (**C**) Average proportion of single and double spots for each cytokine/cytokine combination per total number of spots. PBMC with responses below 40 spots in total were excluded for the analysis (1 donor for CA, 2 donors for TT and 1 donor for PPD) to avoid an impact of subtle differences in spot numbers in the low responders.

Using triple FluoroSpot, the cytokine profile of PBMC from eight donors was investigated after the cells had been stimulated with CA, TT or PPD (Figure 3). A majority of PBMC donors can be expected to respond to these antigens after exposure to commensal fungi (CA), non-pathogenic or pathogenic mycobacteria (PPD), or after vaccination (TT and PPD). All PBMC donors responded to the antigens to different degrees, with some displaying the highest reactivity against CA and others against TT or PPD. All antigens elicited single positive spots representing all three cytokines, as well as lower frequencies of all possible combinations of double positive spots, whereas triple positive spots were rare (Figure 3A). All PBMC donors displayed a higher proportion (*i.e.*, % of total spots) of double- and triple-stained spots after stimulation with CA compared to TT or PPD (Figure 3B). The triple spots were few and found at mean proportions of 0.2%–0.4% of the total number of spots. Despite the limited number of PBMC samples analyzed and variations between them, some patterns in the cytokine profiles were seen (Figure 3C). With regard to single positive spots, CA had a higher proportion of IL-17A and IL-22 while TT and PPD had higher proportions of IFN-γ and IL-22. CA also elicited a higher proportion of double-stained IL-17A/IL-22 spots compared to other double combinations induced by CA, as well as by the other antigens.

**Figure 4:**
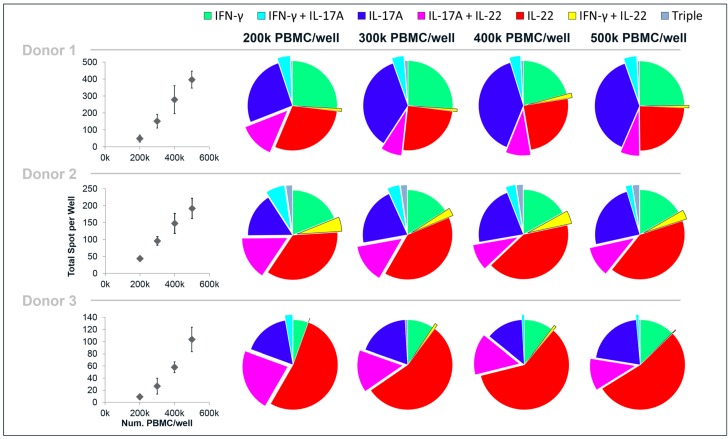
Linearity of PBMC responses to CA, analyzed by IFN-γ/IL-17A/IL-22 triple FluoroSpot. PBMC from three donors were incubated with stimuli or medium alone and analyzed for spots representing cells secreting single-, double- and triple combinations of IFN-γ, IL-17A and IL-22. Quadruplicate wells with 200,000, 300,000, 400,000 or 500,000 PBMC/well were analyzed. The graphs to the left show the mean total number of spots/well and standard deviation obtained for each cell concentration. The charts to the right show the proportion of single-, double- and triple spots for each cytokine/cytokine combination per total number of spots.

The linearity of the cytokine response to one of the antigens (CA) was assessed by incubating different cell concentrations in the wells, from 500,000 down to 200,000 PBMC/well (Figure 4). Decreasing the cell concentrations in the wells had little impact on the cytokine profile in terms of the proportion of single-, double- and triple-secreting cells. Even with as few spots as 10 spots/well (donor 3:200,000 PBMC/well), a cytokine pattern similar to the higher cell concentrations was observed (it should be noted that 10 spots is the mean of a quadruplicate and hence the analysis is based on the average of 40 spots). As could be expected, decreasing cell concentrations led to a decrease of the number of total spots/well. The decrease in spots was proportionally larger than the decrease in cell numbers, suggesting that cytokine responses to the antigens are favored by a high cell density.

### 3.5. Reliability of the Analysis of Double and Triple Spots

**Figure 5:**
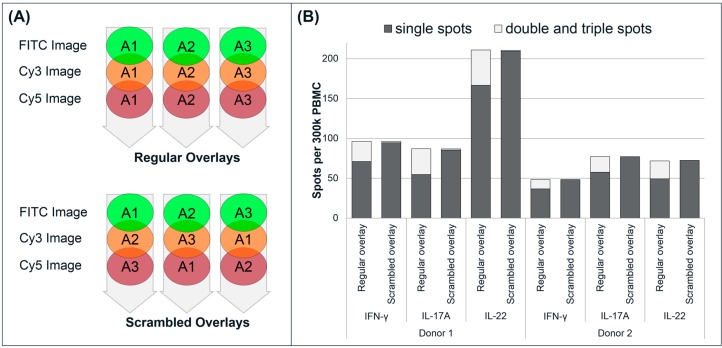
Scrambled analysis of triple FluoroSpot. (**A**) The regular overlay analysis of a well is made by comparing the location of spots on three images (one for each fluorophore) from one well. In the scrambled analysis, three images (each representing one fluorophore) from different wells within a triplicate were mismatched. Thus, one fluorophore image from well 1 was combined with another fluorophore image from well 2, a third fluorophore image from well 3, *etc.*; (**B**) Example of two triplicates analyzed by a regular and scrambled overlay analysis.

To assess the potential risk of having false positive double spots caused by random co-localization (spots generated by adjacent cells), well images from sample triplicates were scrambled (*i.e.*, mismatched) and evaluated for dual and triple secretion (Figure 5) as previously described by Rebhahn *et al.*, 2008 [17]. In the examples shown, control wells represent the regular overlay analysis where a substantial number of double- and triple-stained spots are found (approximately 15% of total events), in addition to the single-stained spots. A scrambled overlay analysis, *i.e.*, of mismatched images from different replicates within a triplicate, only one or two (false positive) double stained spots/well and no triple stained spots could be found.

### 3.6. Potential Impact of Non-T Cell Activation on Antigen-Specific IFN-γ/IL-17A/IL-22 Responses

**Figure 6:**
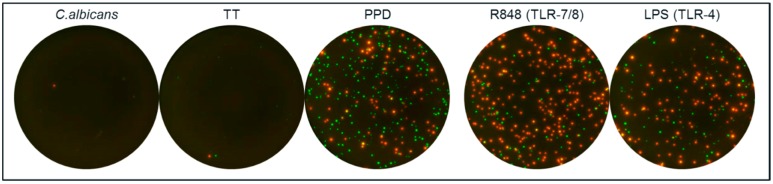
Assessment of the ability of CA, TT and PPD to induce activation of non-T cells. PBMC from two donors were stimulated with CA, TT and PPD and analyzed by a dual IL-6/IL-1β FluoroSpot. TLR agonists R848 (TLR-7/8) and LPS (TLR-4) were included for comparison. Two thousand five hundred PBMC per well were incubated with antigen or TLR agonist and IL-1β and IL-6 detected. IL-1 β spots are shown in red and IL-6 spots in green. PPD and R848 yielded >250 of IL-1β, IL-6 and IL-1b/IL-6 spots per 2500 PBMC and LPS resulted in >150 spots/2500 PBMC. In contrast, PBMC incubated with CA, TT or medium alone (not shown) resulted in a total number of <6 spots/2500 PBMC. The experiment was repeated with other PBMC donors with similar results.

PPD contains molecules that may activate other cells than antigen-specific T cells, and may therefore trigger the release of other cytokines that influence the T-cell response. CA, also a cellular extract, could have similar effects. The antigens used herein were therefore analyzed for induction of cytokines known to be secreted by activated monocytes in parallel with R848 (Toll Like Receptor (TLR) 7/8 agonist) and lipopolysaccharide (LPS; TLR-4 agonist) stimulation. Notably, PPD elicited IL-1β and IL-6 responses comparable to induction seen with R848 and LPS (Figure 6). CA and TT did not and the responses to these antigens are more likely to represent conventional T-cell responses in the absence of strong simultaneous activation of non-T cells.

### 3.7 Discussion

This study shows the functionality of the FluoroSpot assay for the enumeration of human PBMC secreting IFN-γ, IL-17A or IL-22, as well as cell populations secreting combinations of the three cytokines. Most antigen-reactive cells secreted only single cytokines whereas the proportion of cells secreting two cytokines was lower and yet lower for triple-secreting cells. The antigens were selected based on previously shown reactivity with Th1, Th17, and/or Th22 cells upon stimulation of peripheral blood cells or CD4+ T cells from healthy individuals [19,20,21]. In line with this, most donors responded well to the antigens. On average CA induced higher single IL-17A and IL-22 responses than IFN-γ whereas TT and PPD were more prone to induce IFN-γ and IL-22. CA also induced a significantly greater proportion of co-secreting cells than did TT and PPD, in particular IL-17A/IL-22. Stimulation with CA has been shown to elicit CD4+ T cells that are IFN-γ+, IL-17+ or IL-22+ in flow cytometry but also, to a lesser extent, cells co-expressing combinations of these cytokines [19]. In that study, responses to TT were defined as primarily single IFN-γ+. However, increased IL-17A mRNA after stimulation of PBMC with TT has been described [20]. Herein, TT was found to elicit IFN-γ, IL-17A, and IL-22. Discrepancies between studies may be explained by differences in secretion kinetics for the three cytokines. The FluoroSpot, measuring cumulatively over the entire 44-hour incubation, differ in that sense from flow cytometry. Shortening the cell incubation time in the FluoroSpot to 20 h resulted in lower spot counts, in particular for IL-17A. Although IFN-γ and several other cytokines often are analyzed in, e.g., ELISpot after 20 h, the simultaneous analysis of three cytokines in FluoroSpot requires the incubation time to be adapted to suit all three cytokines.

PPD elicited secretion of IL-1β and IL-6 in the same manner as did two TLR agonists known to activate monocytes to secrete these cytokines [22]. This was not seen with CA and TT. This raises the question whether monocyte-derived cytokines influences the PPD-specific T-cell response *in vitro*. When PBMC from healthy donors were stimulated with PPD and analyzed by flow cytometry, a similar cytokine profile as observed in this evaluation was found [21]. In that study, to assess if the T-cell response to PPD was affected by other cells, PPD was substituted with synthetic peptides corresponding to mycobacterial antigens but no shift in the cytokine profile was seen [21]. Nonetheless, caution has to be taken when using antigen extracts for the analysis of T cells.

FluoroSpot is a versatile tool for studying co-secretion of molecules but is also used for the detection of antibodies of different immunoglobulin (Ig) isotypes secreted by B cells that produce only one Ig isotype per cell [23]. Studies utilizing cytokine FluoroSpot cover a variety of cytokines secreted alone or in combinations by T cells and monocytes [16,22]. Several studies have specifically addressed co-secretion of IFN-γ and IL-2 by T cells as an indicator of poly-functionality [24,25,26]. In future studies using IFN-γ/IL-17A/IL-22 FluoroSpot it is likely that both distinct single-secreting cells and co-secreting cells will be found and the ability of FluoroSpot readers to distinguish between spot types is crucial. As shown by the cell titration in Figure 4, the identification of spot sub-populations is insignificantly affected by the total number of spots per well, so long as an accurate spot count is possible. The computerized overlay analysis used herein to define double- and triple-stained spots appears to be precise judging from the near total loss of such spots when mismatched images from different wells were subjected to a scrambled overlay analysis. The coincidence limit (CL) used for these experiments equates to approximately 15 µm. Estimating that the size of resting lymphocytes is approximately 10 µm, false double spots are theoretically possible as two separate single-secreting cells, each producing a uniform spot of one cytokine only, would fit within the CL. However, as only a very small fraction of the cells respond to antigen-specific stimulation, the probability that two single secreting cells lie adjacent to one another is quite small. For the same reason, false triple-stained spots are even less likely. Assessing the probability of false double-positive spots by analysis of scrambled images is particularly important if high spot numbers coincide with low frequencies of double-stained cells.

When capture mAbs to different cytokines are coated in the same well, the absorption of one cytokine may influence the production of another cytokine. In particular this appears to be the case for IL-2. The capture of IL-2 had a negative effect on, e.g., IFN-γ and IL-5 measured simultaneously in dual ELISpot [27]. To compensate for the absorption of IL-2, antibodies to CD28 can be added to the cells and by providing a co-stimulatory signal restore the production of, e.g., IFN-γ [24]. When addressing this potential issue in the IFN-γ/IL-17A/IL-22 FluoroSpot, no absorption effects were seen and no anti-CD28 antibodies were used.

Taking the ELISpot-based technique a step closer to multiplex analysis by utilizing fluorophores for the detection makes the method more comparable to flow cytometry. Still, the methods differ in that flow cytometry detects intracellular cytokines and simultaneously defines the surface markers of the involved cell populations. ELISpot and FluoroSpot, on the other hand, detect antigen-specific cytokine-secreting T cells with a higher sensitivity and are more adaptable to screening of large sample numbers in, e.g., vaccination trials [28,29]. The detection sensitivity of the single FluoroSpot for IFN-γ, IL-17A, and IL-22 was comparable to ELISpot with a slightly better detection displayed by the ELISpot with regard to the low frequencies of spontaneously secreting cells. From the fact that single- and triple FluoroSpot displayed a fully comparable sensitivity, it can be deduced that also the triple FluoroSpot is comparable to ELISpot in terms of detection sensitivity. Many parameters affect the sensitivity of the FluoroSpot assay and there is room for further improvements of tag/anti-tag systems, fluorophores, fluorescence enhancers and the automated readers. The use of tag/anti-tag systems has the benefit that it can be adapted to a variety of assays and inclusion of a fourth tag system allows for future analysis of four cytokines. FluoroSpot amplification strategies, based on primary detection antibodies from different species, followed by secondary detection with species-specific anti-Ig antibodies coupled with fluorophores, have also been used [17]. However, this approach is limited in the sense that it prevents the use of capture antibodies from certain species and requires compatible detection antibodies from different species for each application.

Facilitating a rapid and simple way of determining the relative frequency of cells secreting IFN-γ, IL-17A and IL-22 and intermediate cell populations is likely to help in shedding light upon the nature of immune reactions involved in protective, as well as detrimental reactions.
